# Weighing in on the role of brown adipose tissue for treatment of obesity

**DOI:** 10.3389/jpps.2024.13157

**Published:** 2024-07-17

**Authors:** Brinda Prapaharan, Micah Lea, Jacqueline L. Beaudry

**Affiliations:** Temerty Faculty of Medicine, Department of Nutritional Sciences, University of Toronto, Toronto, ON, Canada

**Keywords:** obesity, energy expenditure, white adipose tissue, brown adipose tissue, weight loss

## Abstract

Brown adipose tissue (BAT) activation is an emerging target for obesity treatments due to its thermogenic properties stemming from its ability to shuttle energy through uncoupling protein 1 (Ucp1). Recent rodent studies show how BAT and white adipose tissue (WAT) activity can be modulated to increase the expression of thermogenic proteins. Consequently, these alterations enable organisms to endure cold-temperatures and elevate energy expenditure, thereby promoting weight loss. In humans, BAT is less abundant in obese subjects and impacts of thermogenesis are less pronounced, bringing into question whether energy expending properties of BAT seen in rodents can be translated to human models. Our review will discuss pharmacological, hormonal, bioactive, sex-specific and environmental activators and inhibitors of BAT to determine the potential for BAT to act as a therapeutic strategy. We aim to address the feasibility of utilizing BAT modulators for weight reduction in obese individuals, as recent studies suggest that BAT’s contributions to energy expenditure along with Ucp1-dependent and -independent pathways may or may not rectify energy imbalance characteristic of obesity.

## Introduction

Since the identification of metabolically active BAT in adult humans in 2009 [1], there has been a growing interest towards the idea of BAT activation as a therapeutic strategy to assist in treating obesity. Obesity, defined as excessive fat storage, is associated with increased risk of cardiovascular diseases and metabolic disorders including type 2 diabetes (T2D) and dyslipidemia. Consequently, finding a treatment to mitigate the development of obesity and its related diseases is crucial. Obesity is characterized by an energy imbalance, where energy intake exceeds energy expenditure (EE) [2]. Adipose tissue, a vital and dynamic organ, provides structural support but can be influenced by external and internal signals that affect its function. BAT has emerged as a potential therapeutic target for obesity due to the presence of uncoupling protein 1 (Ucp1), a thermogenic protein that boosts EE within the body [3]. Ucp1 is triggered by sympathetic activity and facilitates thermogenesis by redirecting proton flow in the BAT inner mitochondrial membrane to generate heat rather than ATP [3]. Ucp1 can also be present in white adipose tissue (WAT), where it is involved in a process known as “browning”. White adipocytes with Ucp1 develop a BAT-like phenotype including an increase in mitochondria, and formation of smaller lipid droplets within the cell. Enhancing BAT activity and browning in WAT could optimize EE, potentially restoring energy balance in obese individuals. To combat obesity by utilizing BAT activation mediated increased EE, it is essential to understand the mechanisms that govern BAT activation and inhibition, in both rodents and human models. Activators such as cold-exposure, thyroid hormones, and fish oil-derived omega-3 fatty acids promote BAT activity by upregulating the gene expression of Ucp1 and other browning-associated proteins [4–6]. Conversely, glucocorticoids, androgens, aldosterone, and high fat diet serve as BAT inactivators and hinder thermogenic function by dysregulating adipocytes and inflammation [7, 8]. It is imperative to study BAT in humans, which is known to vary in abundance, particularly in obese individuals [9]. Additionally, assessing the function of Ucp1 in BAT thermogenesis is also essential. In recent years, Ucp1 has been acknowledged as one of several thermogenic pathways in BAT, alongside newly identified Ucp1-independent pathways, specifically calcium and creatine substrate cycling [10, 11]. Consequently, the objective of this review is to compile preclinical and clinical evidence identifying pharmacological, hormonal, bioactive, and environmental factors that modulate BAT activity through both Ucp1-dependent and independent mechanisms. Endogenous factors that influence BAT activity and induce browning are analogous; however, the outcomes from exogenous factors and stimuli that modulate adipose tissue function and phenotype can differ [12]. Thus, the importance of browning in regulating EE, glucose, and lipid homeostasis should not be overlooked. Our primary focus is to review and discuss the potential of BAT as a viable therapeutic target for combating obesity, and present factors influencing BAT activation rather than WAT browning.

## Activators of brown adipose tissue

### Pharmacological activators

In both rodents and humans, BAT is activated by sympathetic nervous system (SNS) stimulation [13] through the release of norepinephrine and its non-selective agonism on β1, 2, and 3 adrenergic receptors (ADRs) on various BAT tissues [14]. Downstream activation of the βADRs increases the release of stored nutrients such as free fatty acids that upregulate Ucp1 in BAT and can also induce browning in WAT. Moreover, recent research indicates that Ucp1 is dispensable to increasing EE due to browning through the Ucp1 independent futile creatine cycling [15]. While stimulation by β3 agonists activates BAT in rodents [16, 17], there is conflicting evidence regarding which βADR subtype is the primary activator of human BAT [18–21]. In both rodents [22] and humans [23], BAT activation through cold exposure increases fatty acid uptake through the activation of βADRs. Obese and T2D individuals, who could benefit from increased EE through BAT activation, generally lack the presence of metabolically active BAT. Nevertheless, both rodent and human studies have investigated several pharmacological agents that activate BAT, through various mechanisms, including direct and indirect βADRs. However, these studies have shown differing levels of success. In the next section we will review BAT activators and discuss the relevance of the pathways involved.

### Evidence in rodents

Various pharmacological activators have been studied in rodents to upregulate BAT activity for weight loss purpose. The activation of BAT results in the maintenance of thermal homeostasis and disposal of excess metabolites [1]. 2-4-dinitrophenol (DNP), a component of explosives, wood preservation solutions, and herbicides, was commercially used as a weight loss medication for 5 years in the 1930s [24]. While not a direct activator of BAT, DNP induces extreme increases in metabolic rate and thus weight loss through widespread mitochondrial uncoupling [24]. Interestingly, these mechanisms appear to be independent to Ucp1 pathways. DNP supplemented drinking water increased metabolic rate and induced fat and total body mass loss in diet-induced obese (DIO) female C57BL/6J mice held at thermoneutrality (30°C) after 9 weeks [25]. Surprisingly, DNP treatment decreased mRNA and protein levels of Ucp1, which could be reflective of reduced BAT function even in the face of increased EE.

Berberine (BBR), a plant derived anti-obesity medication, increases adipose triglyceride lipase (ATGL) expression and basal rates of TG lipolysis in adipose tissue [26]. Daily intraperitoneal (i.p.) BBR administration of 1.5 mg/kg for 6 weeks to obese C57BL/6 male mice increased BAT thermogenesis and EE [27]. Interestingly, this BBR treatment also increased BAT volume, glucose uptake, and *Ucp1* and *Prdm16* expression levels in these obese male mice [27]. Similarly, 4 weeks of BBR i.p. injections at a higher dose of 5 mg/kg/day in obese male C57BL/6J mice increased EE, lipid oxidation, BAT ^18^F-FDG uptake, BAT mitochondrial content, UCP1 protein expression, and BAT transcription factor genes such as peroxisome proliferator-activated receptor gamma coactivator 1-alpha (*Pgc1-α*) that regulate energy metabolism [28]. This indicates that BBR can induce BAT activation through mechanisms other than direct βADR agonism. However, the direct pathways through which BBR increases BAT remains unclear. Other plant derived agents include *Withania somnifera* extract (WSE), and withaferin A (WFA) a major constituent of WSE. Dietary supplementation of 0.5% WSE to a HFD for 10 weeks increased mRNA expression of genes involved in thermogenesis and mitochondrial biogenesis, and reduced lipid droplet size in BAT of DIO male C57BL/6J mice [29, 30]. Oral supplementation (1.5 mg/kg) of WFA for 7 days in DIO male C57BL/6J mice increased EE, UCP1 protein and mRNA levels in BAT, and phosphorylation of p38 and ERK_1/2_ in WAT indicative of browning. Interestingly, WFA treated mice closely resembled the phenotypic results observed in the metabolically healthy low-fat diet-fed group [30]. While not providing direct evidence of BAT activation, these studies suggest that WSE and WSA may influence mitochondrial biogenesis and Ucp1 expression in both BAT and WAT.

Resveratrol, a polyphenol produced in the skin of fruits such as grapes and berries, has been investigated for its protective effects against weight gain [31, 37] and obesity related diseases. It has been extensively reviewed [32–35]. However, in brief, the changes in adipose tissue are suggested to act through WAT browning by promoting AMPK phosphorylation, which increases PGC1α and sirtuin 1 (SIRT1) production. This induction leads to mitochondrial biogenesis and browning, rather than direct BAT activation [36, 37]. This is supported by morphological changes in WAT to BAT-like lipid droplets, and increased BAT mass [38]. Alternately, resveratrol may adjust gut microbiota composition that improves intestinal barrier dysfunction and reduces inflammation [39]. While resveratrol supplementation offers benefits in improving metabolic conditions, there is little evidence supporting direct BAT activation. The interaction between resveratrol and adipose tissue appears to occur through WAT browning rather than BAT activation.

Several studies have evaluated βADR agonists to increase BAT activity in rodents. BAT in rodents expresses functional β3 and β1 ADR, with β3ADR now being well-established to directly upregulate BAT processes [40]. In obese female C57BL/6 (*ob*/*ob*) mice, oral administration of β3ADR agonists BRL 26830A, BRL 33725A, and BRL 35135A for 4 weeks showed a decrease in weight gain with no change in food intake, and BAT UCP1 protein expression [41].Wilson S et al reported that lean male Sprague-Dawley rats given single i.p injections of 5 mg/kg of BRL 26830A increased EE while simultaneously increasing lipid oxidation in BAT, thus resulting in a substantial decrease in BAT lipid droplet size [42]. In sedentary obese male Zucker rats, gastric cannula administration of BRL 35135 increased mitochondrial guanosine diphosphate (GDP) binding, an indicator of increased Ucp1 uncoupling, mitochondrial protein content, and reduced WAT mass compared to those that received either vehicle or an ⍺_2_ ADR agonist [43]. Both acute and chronic treatment with the selective β3ADR agonist ICI D7114 also suggest BAT activation in rats. In male Wistar rats, ICI D7114 increased EE and mitochondrial GDP binding in BAT suggesting BAT activation, however this was associated with an increase in heart rate [44]. As chronic tachycardia increases risk of cardiovascular events, a human safety trial is warranted [45]. To note, ICI D7114 had no effect on weight loss or changes in fat mass in male Sprague-Dawley rats [46]. Due to technological limitations in these studies, it is difficult to attribute metabolic changes to BAT activation alone.

Recently, there has been a shift towards investigating non-sympathomimetic methods for BAT activation, as sympathomimetics could inadvertently stimulate the central nervous system and non-target organs. Peroxisome proliferator-activated receptor γ (PPARγ) is a transcriptional regulator necessary for the maturation of brown adipocytes [47]. It promotes BAT adipocyte differentiation and lipid storage [48]. PPARγ agonists have been used in T2D treatment to improve insulin sensitization but can result in weight gain. Interestingly, PPARγ mediated increases in oxygen consumption and fatty acid oxidation have been repeatedly shown *in vitro* [49, 50]; however, these effects are not observed *in vivo*. In fact, opposite effects are seen in both humans [51] and rodents [52], where PPARγ activation increases lipid storage. While PPARγ agonism is an exciting concept for BAT activation *in vitro*, the inability to replicate increases in EE and lipid oxidation *in vivo* makes its use in treating metabolic complications beyond insulin sensitization unlikely.

Signalling from the angiotensin II (AngII) has regulatory effects on energy homeostasis and can act through PPARγ activation to promote adipocyte differentiation and insulin sensitivity. However the role of AngII signalling specifically in BAT is less clear [53]. Recently, the use of angiotensin type 2 receptor (AT2R) agonism has been explored as an alternate method of BAT activation [54]. C21, an AT2R agonist, was not protective against weight gain from HFD feeding in male C57BL/6J mice. However, C21-treated mice showed increased BAT mass and brown adipocyte differentiation despite being fed a HFD compared to mice fed a regular chow and treated with C21. While direct measures of BAT activity were not taken, protein levels of UCP1 and electron transport chain complexes I, II, III, and IV were upregulated with C21 treatment in HFD fed mice independent to changes in ATP synthase. This may suggest increased thermogenic capacity in an obese state, however further investigation is required to determine if AT2R agonism can improve metabolic outcomes such as glucose or lipid levels and if there is indeed BAT activation.

Metabokines produced from branch chain amino acid catabolism including 3-methyl-2-oxovaleric acid (MOVA), 5-oxyproline (5OP), and β-hydroxyisobutyric acid (BHIBA) significantly increase the expression of genes associated with BAT thermogenesis and lipid metabolism [55]. DIO mice treated with either MOVA, 5OP, or BHIBA via drinking water for 17 weeks showed increased EE with no changes in overall activity or food intake. Additionally, these metabokines showed higher levels of mitochondrial biogenesis markers and ^18^F-FDG uptake in BAT. MOVA and 5OP given together reduce fat mass by nearly 25% and improve glucose tolerance. The TCA cycle intermediate succinate is another potential metabolic signal of BAT activation [56]. Obese mice provided with succinate supplemented drinking water for 4 weeks had markedly decreased body weight, and improved glucose tolerance independent of changes in food intake. This response appears to be driven through Ucp1 mediated thermogenesis as *Ucp1* KO mice abolishes this positive effect. These results highlight the potential of non-sympathomimetic BAT activation.

### Evidence in humans

Sympathomimetics are known to increase EE, however it is unclear if this is mediated by BAT activation in humans. Ephedrine, approved for treating hypotension, exerts its effects by inhibiting neuronal reuptake of norepinephrine allowing more time for it to act on postsynaptic βADRs [57]. Acute oral ingestion of 1 mg/kg body weight ephedrine in fasted healthy men increased perirenal BAT blood flow after 30 and 60 min in some participants [58]. This corresponded with an increase in both perirenal BAT and overall body temperature, circulating glucose and oxygen consumption by 19% after 60 min, and reduced RER after 150 min with no change in circulating non-esterified fatty acids (NEFAs) or glycerol levels. Both systolic blood pressure and heart rate increased over the duration of the experiment. A higher dose of 2.5 mg/kg body weight of ephedrine increased ^18^F-FDG uptake into supraclavicular BAT in lean fasted men, but not in obese or placebo groups [19]. This also corresponded with increased BAT activation in lean but not obese participants and resulted in no change in core body temperature or plasma NEFA levels. Ephedrine also increased EE and circulating glucose, but also increased systolic blood pressure. In contrast to acute treatment, chronic treatment with 1.5 mg/kg ephedrine orally for 28 days in fasted metabolically healthy men results in no change in resting EE, respiratory exchange ratio (RER), and a decrease in systolic blood pressure and BAT activity at the end of treatment [59]. Cypess et al reported that lean male and females that received an intramuscular injection of 1 mg/kg ephedrine and subjected to cold exposure (14°C) maintained their temperature without shivering [60]. Both cold and ephedrine increased metabolic rate by 79 and 136 kcal/day, respectively, and decreased RER indicating more use of fatty acids as fuels, but no changes were observed between interventions. Systolic and diastolic blood pressure increased with cold and ephedrine treatment but only ephedrine increased heart rate [60]. Ephedrine elevated plasma glucose, NEFA, lactate, and insulin levels and no change in ^18^F-FDG uptake compared to cold and placebo groups. Remarkably, this study included female participants and no sex-based differences were reported in response to ephedrine administration.

While the reasoning for using ephedrine as a BAT activator is logical, these results are conflicting and may suggest shifting focus to other approaches. With increases in EE and BAT activity, heart rate and blood pressure also increase with ephedrine. This makes the individual contribution to BAT difficult to discern as a systemic increase in blood flow and nonspecific βADR activation could account for much of this increase [18]. Additionally, the inability for ephedrine to stimulate BAT activity in obese individuals and its reduction in potency of BAT stimulation with chronic treatment make its use in treating obesity unlikely. It is also important to consider the extent of EE afforded by treatments that would lead to meaningful weight loss in obese individuals. Although ephedrine increased EE, this minimal increase may not be clinically relevant. Moreover, individuals with obesity did not respond to ephedrine for reasons unknown thereby rendering ephedrine an unlikely candidate to expend excess stored calories in humans.

Other approaches include direct βADR agonists that mimic SNS stimulation. Mirabegron, a β3ADR agonist used to treat overactive bladder [61], has been explored as a sympathomimetic BAT activator. Higher than the approved dose for hyperactive bladder, which is 50 mg, mirabegron shows promising effects to upregulate BAT activity. Healthy men selected with cold induced detectable BAT given an acute dose of 200 mg mirabegron showed an increase in BAT activity measured by ^18^F-FDG uptake in all 12 participants [62]. Compared to placebo, there was higher resting metabolic rate, plasma glucose and NEFA, heart rate, and systolic blood pressure. However, these cardio stimulatory effects were lower compared to ephedrine [19, 60]. Interestingly, there were no changes in ^18^F-FDG uptake in subcutaneous WAT, liver, or skeletal muscle, except for BAT that showed detectable changes in ^18^F-FDG uptake with mirabegron treatment. A dose dependent effect on BAT activation was found when comparing between 50 and 200 mg mirabegron in healthy young men with cold activated detectable BAT [20]. Of note, only the 200 mg dose increased EE in these men with no changes in plasma glucose or insulin and increases in NEFA levels were dose dependent possibly through β3ADR mediated lipolysis. Heart rate and systolic blood pressure were higher with the 200 mg dose. 150 mg/day mirabegron treatment for 10 weeks increased UCP1 and CIDEA protein expression in subcutaneous WAT to a greater extent than cold treatment, however there was no change in PCG1α, a known indicator of mitochondrial biogenesis or mitochondrial DNA content, which is activated by βADR in rodents [63]. No change in heart rate or blood pressure were seen indicating that cardiovascular effects may only be an acute side effect. Chronic treatment with 100 mg mirabegron for 4 weeks increases BAT activity and volume in healthy women, particularly in those with initially lower BAT amount [64]. While there were acute changes with EE and RER, after 4 weeks there were no differences from baseline. At this lower dose, heart rate and systolic blood pressure on day 1 were higher to a greater degree than in subjects from previous studies [20, 62, 63]. The increase in NEFA levels after chronic mirabegron treatment was decreased compared to day 1 and increased fasting plasma high density lipoprotein content after 4 weeks suggesting alterations to whole body lipid metabolism. In lower doses, mirabegron did not elicit any effects on BAT activation, however the higher 100–200 mg dose cause an increase in BAT activity. Similar results were shown with acute 50 and 200 mg mirabegron increased EE, however, only 200 mg increased BAT blood flow, oxidative metabolism, and ^18^F-FDG uptake [18]. While β3ADR signalling is assumed to activate BAT, 50 mg dose showed no change in BAT activity. Similar to previous observations, higher doses of mirabegron increased heart rate and systolic blood pressure, which indicate nonselective βADR agonism by mirabegron and alternative βADR subtype activation of BAT [18]. Contrasting results have emerged regarding the abundance of βADR subtype in human BAT. β3ADR is reported as being the most abundant followed by with β2ADRs *in vivo* [65, 66]. Conversely, others report almost non-existent β3ADR mRNA expression *in vitro* in human BAT [67]. Differentiated human BAT adipocytes *in vitro* treated with formoterol, a highly selective β2ADR agonist, showed a similar increase in oxygen consumption rate (OCR) as norepinephrine, and higher than mirabegron [18]. Additionally, this effect was inhibited when formoterol was used in conjunction with a β2ADR antagonist. *In vivo*, formoterol has been shown to increase resting EE, and decrease RER with no change in heart rate or blood pressure in lean men, however no measures of BAT activity were performed [68]. β2ADRs have been shown to increase EE and rates of lipolysis in non-human models [69], however, if human BAT is indeed activated by β2ADR agonism, these alterations of EE and RER could potentially be mediated by BAT. However, these studies have yet to be explored.

Direct sympathomimetics effectively increase BAT EE but show limited efficacy in treating obesity by upregulating BAT thermogenesis. Mirabegron increases EE and BAT activity, but its cardio stimulatory effects could potentially be dangerous for certain populations. Notably, females and other target demographic of overweight and obese individuals who are already at a higher risk of hypertension and other cardiovascular complications.

### Hormones

#### Evidence in rodents

##### Fibroblast growth factor 21

Fibroblast growth factor 21 (FGF21) is a hormone produced by the liver, which is involved in both glucose and lipid homeostasis [70, 71]. FGF21 is secreted during the fasting state and upon binding to FGF receptors (FGFR) and ß-klotho (KLB) on target tissue, increases expression of Pgc-1α, a key component of energy metabolism [72]. In turn, this increases fatty oxidation, glucose production and transcription of Ucp1 in adipose tissue to improve thermogenic capacity [73]. To date, the effects of FGF21 as a browning agent of WAT and BAT have been largely seen in rodents and are less evident in humans. In adult male Siberian hamsters, FGF21 treatment of 3 mg/kg for 7 days increased glucose and lipid uptake in interscapular BAT (iBAT) and glucose uptake in subcutaneous WAT (sWAT) and visceral WAT (vWAT) compared to pair-fed controls [74]. Treated mice experienced greater weight loss independent of food intake, increased EE, reduced RER, and increased *Ucp1* mRNA expression in iBAT, sWAT and vWAT [75]. Weight loss and browning effects of FGF21 persist in obesity as DIO male mice treated daily with FGF21 (1 mg/kg) for 2 weeks experience a 20% reduction in body weight without affecting food intake, and upregulated gene expression of *Pgc-1α*, and *Ucp1* in WAT and BAT compared to controls [76]. Similarly, HFD fed mice treated with 30 mg/d for 5–7 days see increased body temperature and greater EE [76]. The effectiveness of FGF21 also appears to be dependent on the presence of FGFR and KLB on adipocytes [77]. Paradoxically, obesity is associated with an increase in serum FGF21 levels and attributed to a reduction in FGFR and KLB expression on adipocytes [78, 79]. Overexpression of KLB in adipose tissues shows protection against DIO with increased *Ucp1* expression in WAT and lower plasma FGF21 levels compared to WT counterparts [80]. Whereas removing KLB from iBAT, iWAT and eWAT reduced *Ucp1* mRNA and protein expression and cold tolerance compared to WT control mice [78]. Therefore, in mice, FGF21 administration and receptor expression in adipose tissue seems to regulate EE and BAT activity.

#### Evidence in humans

Similar to rodents, both male and females that suffer from obesity exhibit higher levels of serum FGF21 compared to lean counterparts [1, 23], suggesting FGF21 resistance with increased levels of adiposity [79]. This increase in circulating FGF21 levels is generally attributed to a reduction in KLB expression in vWAT and sWAT [81]. Polymorphisms of the KLB gene are associated with obesity [82], further demonstrating the vital role of this receptor in facilitating FGF21 induced-weight loss. Clinical trials on FGF21 analogues demonstrate weight loss in obese individuals however causal relations with thermogenesis were not investigated. In a cohort of obese and T2D subjects (n = 38) FGF21 analogue LY2405319 modestly reduced weight in the high dose group (20 mg for 28 days) [83], but no measurements of food intake and EE were taken. More recently, the analog LL580 was found to have no effect on body weight in 64 obese participants across 12 weeks of treatment [84], but again no measures of thermogenesis were taken. Furthermore, safety profile of this analogue requires comprehensive evaluation as subjects in the trial developed serious adverse effects including lymphoma and respiratory failure [84]. More studies are needed to assess the pharmacological efficacy of FGF21 administration in humans.

### Irisin

#### Evidence in rodents

Irisin is a hormone secreted by skeletal muscle and adipose tissue [85]. Irisin is generated from the cleavage of FNDC5, a protein produced in response to transcriptional co-activator Pgc-1a [86]. Irisin released from the skeletal muscle into the bloodstream upon exercise stimulates WAT browning by increasing the expression of *Ucp1* encoding genes [87]. This process of irisin promoting Ucp1 expression happens downstream upon its binding to the adipocyte surface and is facilitated through the extracellular signal related kinase (ERK) and p38 mitogen-activated protein kinase (p38 MAPK) signalling pathways [88]. Previous research has identified how irisin can promote browning of white adipocytes in obese models however recent reports have critically examined the translation of research in rodents and potential of irisin in WAT in humans [89]. HFD fed mice given a daily dose of irisin (0.5 μg/g) for 2 weeks reduced body weight and significantly increased expression of browning genes including *Ucp1*, *Pgc-1α*, positive regulatory domain containing 16 (*Prdm16*), and transmembrane protein 26, which is a marker of beige cells in WAT [88]. However, no functional assessments were conducted to measure any impact on thermogenic capacity besides UCP1 protein content [88]. A significant limitation of recent research is a dearth of established reference values for endogenous irisin content in rodents or humans, which exhibit variable ranges spanning from 0.3 ng/mL as measured using mass spectrometry to 50–900 ng/mL measured using ELISA [89]. Although these findings support the proposition that irisin improves thermogenic capacity and promotes browning of WAT, methodological disparities in establishing irisin content severely limit the interpretation.

#### Evidence in humans

Zhang et al demonstrated that in mature human derived adipocytes, irisin treatment induces thermogenesis by upregulating *UCP1* expression in sWAT by upstream activation of the ERK and p38 MAPK pathways, without changing UCP1 or PRDM16 protein content in BAT derived adipocytes [90]. Due to inconsistencies in reporting endogenous irisin levels in humans as well (reviewed in 73), irisin function is indefinite. To note, these studies also do not report changes in *UCP1* expression in WAT of humans following exercise, which is known to stimulate irisin release [91]. Obesity is associated with a significant reduction in gene expression of *FNDC5*, irisin precursor, in muscle and both sWAT and vWAT [92]. However, no differences in muscle and WAT irisin levels have been found between obese and lean participants [93]. Furthermore, meta-analysis has failed to detect substantial evidence supporting a direct relation between irisin levels and disease development due to highly variable circulating irisin levels and absence of validated antibodies (reviewed in [89]). This coupled with limited subject numbers and methodological limitations, renders it uncertain whether irisin can adequately induce BAT activity or browning of WAT in obese humans.

### Thyroid hormones (TH)

#### Evidence in rodents

Thyroid hormones (TH) are prominent modulators of metabolism in the body [94]. TH refers to the hormones thyroxine (T_4_) and its active form triiodothyronine (T_3_), produced and released by the thyroid gland. Exogenous intracerebroventricular delivery of T_3_ increases SNS activity and expression of *Ucp1* mRNA in BAT of rats [95]. In both *ob/ob* and DIO mice, pharmacological activation using GC-1 (synthetic form of T_3_) increased expression of thermogenic genes including *Ucp1, Prdm16, Cidea* in sWAT, but repressed the same genes in BAT [96]. Interestingly, levothyroxine (synthetic T_4_) successfully promoted glucose uptake of BAT in DIO mice following cold exposure, showing conflicting impacts of TH on BAT [97]. HFD fed mice without functional thyroid receptors (TR) in the hypothalamic neurons (*TR* ^hypo−/−^) maintain thermogenic activity following cold exposure (4°C) but have reduced sympathetic activity in BAT and are more susceptible to DIO, highlighting how neuronal TH interactions regulate BAT function [98]. As TH mimetics continue to evolve, there is a growing interest in developing treatments that selectively targets the liver and adipose tissues to decrease ectopic lipid accumulation without adversely impacting the cardiovascular and bone mineralization systems. Some attempts have been made to pair the TH treatment with other peptides such as glucagon that would target BAT while simultaneously promoting liver action to offset hepatic steatosis, hyperglycemia and hyperlipidemia in DIO male mice [99]. The combo of glucagon/T3 produced no change in BAT gene expression profiles but triggered UCP1 gene and protein upregulation in iWAT, however, efficacy of combination was lower than T3 alone. Moreover, glucagon/T3 treatment in *Ucp1* ^−/−^ mice partially reduced changes in EE and RER, suggesting that other pathways besides UCP1 in the adipose tissues remain at play. Furthermore, increased bone turnover, cardiac volume, reduced fractional shortening and ejection fraction, suggest cardiovascular and bone thyrotoxicity of T3 treatment alone, which were mitigated in T3/glucagon combination [99]. Despite these beneficial effects in the mouse model, intensive investigation in humans is warranted as the glucagon receptor is less expressed in adult adipose tissue and the pharmacological amount required to induce enough “beiging” of WAT to upregulate EE remains unknown (reviewed by [100]).

#### Evidence in humans

WAT from obese participants, serum T_4_ levels positively correlated with mRNA levels of *UCP1, CIDEA* and *PRDM16* in both sWAT and vWAT from individuals with obesity [101]. Although research in obese populations is limited, studies conducted in hyperthyroid populations demonstrate that increased TH levels stimulate higher BAT activity, EE and fatty acid oxidation, as evidenced by lower RER levels [102]. Additionally, in euthyroid males, T_3_ levels were inversely associated with BMI and positively associated with greater pericardial fat volume, implying a link between TH and lower BMI as well as greater thermogenic activity [103]. However, the potential adverse effects of TH therapy continue to be a concern. Achieving the desired effects on the liver and adipose tissue without any detrimental consequences on cardiovascular and bone health need further examinations.

### Orexin

#### Evidence in rodents

Orexins (OX) are a group of neuropeptides, including Orexin-A and Orexin-B, generated in the hypothalamus. They were first discovered in 1998 in the lateral hypothalamic regions of the brain, which are associated with the regulation of feeding [104]. OX regulates sleep-wake cycle, arousal and is most importantly involved in the food-reward system by increasing motivation for palatable foods. In addition to these functions, OX is also required for brown adipocyte development. OX-null mice fed a HFD experience rapid weight gain, and OX-null neonates have reduced lipid accumulation and mitochondrial content [105]. These effects are prevented in offspring of OX-null mice given 3 injections of 30 mg/kg of Orexin-A [105]. *In vitro*, OX is necessary for brown adipocyte differentiation by stimulating adipogenesis via p38 MAPK [105]. OX is also involved in the thermogenic effect of bone morphogenetic protein 8B (BMP8B), a batokine released by mature brown adipocytes. BMP8B facilitates thermogenesis in part by inhibiting AMPK in the ventromedial nucleus of the hypothalamus, which increases OX expression in the lateral hypothalamus area [106]. As such, in OX-null mice, BMP8B treatment does not produce any thermogenic effects and in rats via inhibition of VGLUT2, glutamate transporters highly expressed in OX neurons, BMP8B treatment blunts thermogenic effects and decreases expression of UCP1 protein [106]. Overall, this suggests greater regulation of BAT activity through a hypothalamic network, where the influence of OX on BAT is dependent on AMPK activity.

Apart from OX, its receptor, orexin-receptor 1 (OXR1) is also integral to BAT function as an ablation of the receptor in mice leads to reductions in lipid stores in iBAT [105]. In HFD fed mice, inactivation of OXR1 in serotonergic neurons impairs energy homeostasis by reducing glucose uptake, *Ucp1* gene expression, mitochondria function and insulin sensitivity in BAT [107]. Therefore, OXR1 expression in the brain appears to play a protective role in maintaining peripheral glucose metabolism and BAT activity.

#### Evidence in humans

In humans, plasma levels of OX are inversely related to BMI, and significantly lower in obese and morbidly obese individuals compared to normal or overweight counterparts [108]. Moreover, higher serum levels of OX correlate to improved insulin sensitivity and lipid profile [109]. Expression of HRCTR1, the gene encoding OXR1, is found in human adipose tissue. Moreover, the gene is primarily expressed in vWAT rather than scWAT of non-obese males [109]. Investigating the role of OX on BAT activity in the neck and abdominal regions, Pino et al concluded that 100 nM of OX had no impact on BAT gene expression in differentiated cells, despite observing an inverse relationship between BMI and *OXR1* mRNA expression [110]. Additionally, treatment of 100 nM of OX had no impact on *UCP1*, *PPARGC1a*, or *OXR1* mRNA expression [110]. Hence, while studies suggest endogenous OX is greater in normal weight individuals, treatment using OX may not induce beneficial changes to BAT development that would increase thermogenic capacity.

### Dietary

#### Fish oils

##### Evidence in rodents

Fish oils contain high levels of n-3 polyunsaturated fats (PUFAs), specifically eicosapentaenoic acid (EPA) and docosahexaenoic acid (DHA) that the body is unable to synthesize on its own. Fish oils are a well-documented activator of thermogenic activity in adipose tissue and n-3 PUFAs promote anti-inflammation and SNS activity [6]. The addition of EPA (36 g/kg) to HFD (45%) feeding in male C57BL/6J mice for 11 weeks increased UCP1 protein in BAT and other browning marker genes including *Prdm16*, *Pgc-1a* and *Fgf21*, whereas DHA (10 or 50 mg/kg) in HFD (60% fat) feeding over 8 weeks in male mice increased UCP1 content and anti-inflammatory macrophages in eWAT [111, 112]. In aging DIO mice, administration of DHA and EPA (683.4 mg/g, 46.7 mg EPA/g) restored UCP1 content, and increased presence of beneficial pro-resolving lipid mediators that are suggested to reduce age-related inflammation in BAT [113]. In obese mouse models, DHA increases Akt phosphorylation, downstream of the insulin receptor in eWAT and sWAT, demonstrating a positive effect on insulin signalling [112, 113]. Fish oil (DHA 25%, EPA 8%) may target the SNS by activating β3ADR situated on the BAT in lean mice [114], but the effect of DHA and EPA to directly act on BAT in obese rodent models is unknown. The role of lipid mediators, derived from PUFAs present a new class of thermogenic regulators such as DHA derived lipid mediator, maresin 1 (MaR1) [115]. MaR1 promotes glucose uptake, enhances fatty acid oxidation, and upregulates anti-inflammatory and thermogenic gene expression in rodent brown adipocytes. Additionally, it induces M2 phenotype in macrophages and contributes to beige adipocyte remodelling in WAT of DIO mice [115]. In addition, the lipid mediator prostaglandin E_2_ (PGE2), which is synthesized from arachidonic acid, n-6 PUFA, also induces beigeing phenotype in WAT [116]. These noteworthy effects showcase how fish oils through their derived lipid mediators may attenuate inflammation and adipocyte dysregulation in both WAT and BAT.

##### Evidence in humans

REDUCE-IT trial with icosapent ethyl, a highly purified EPA, reported improvement in hypertriglyceridemia and cardiovascular risk reduction [117]. In contrast, other studies showed no improvement in cardiovascular health with n-3 carboxylic acid [118], or a mixture of EPA and DHA [119] compared to corn oil placebo. The addition of 200 µM EPA to subcutaneous adipocytes derived from overweight female subjects resulted in an increase in *UCP1* and *PRDM16* and *CPT1* expression, indicating that EPA may promote fatty acid oxidation and thermogenesis in WAT [120]. These findings have been corroborated in sWAT adipocytes of lean females, suggesting browning by EPA may be independent of body mass [121]. Clinical studies in humans have not identified any direct relationships between EPA, DHA and BAT activity, however, consumption of DHA and EPA over 12 weeks improved insulin resistance in individuals with obesity by reducing fasting insulin levels independent of weight loss [122]. While fish oil can aid in reducing excess fat storage and increasing thermogenic capacity in human adipocytes *in vitro*, the lack of comprehensive *in vivo* studies limits translatability.

#### Bioactives

##### Evidence in rodents

There are a variety of bioactives that may induce browning of WAT or increase thermogenic capacity of BAT in obese rodent models. Quercetin, or onion peel extract (OPE) (50–150 μg/mL) induces browning in white adipocytes and increases mRNA expression of *Ucp1* dose-dependently [123]. The addition of OPEs to HFD fed mice increases fatty acid uptake and expression of thermogenic marker genes in WAT, without changes in body weight [123, 124]. Ginger activates Sirtuin 1 and PGC-1α signalling to induce thermogenic gene expression in both BAT and WAT of HFD mice, consequently reducing body weight and fat accumulation [125, 126]. Ginger capsules (500 mg/kg/d) have been reported to restore citric acid cycle metabolites altered by HFD (60% calories from fat) [126]. Similarly, allicin (garlic extract) induces browning of iWAT in HFD fed mouse models by increasing *Pgc-1α, Prdm16* and *Ucp1* expression [127]. Allicin regulates thermogenic gene expression in white adipocytes by increasing krupper-like factor 15, a transcription factor that regulates *Ucp1* expression via the ERK MAPK signalling pathway [127]. Curcumin and capsaicin are bioactives that have more substantial findings that appear to activate BAT in rodent models. A derivative of turmeric, curcumin (1%) reduces WAT inflammation, increases BAT mRNA expression of *Ucp1,* and improves thermogenic response following cold exposure in HFD (60% fat) fed male mice [128]. Similarly, pure capsaicin (0.01%) increases EE by acting on TRPV1 channels to increase *Ppar-α, Prdm16,* and *Pgc-1α* gene expression to facilitate browning of WAT affording protection against DIO in mice [129]. While these bioactives promote browning, their usage is restricted due to limited bioavailability and challenges in dosing [130–132].

##### Evidence in humans

In female participants with high adiposity, consumption of dried ginger extract (600 mg/d) for 3 months has no impact on EE [133]. Similarly, single consumption of a dried ginger powder capsule in males (1 g), failed to change body temperature [134]. Moreover, capsaicin administration did not elicit change in BAT EE or body weight in both lean and overweight subjects independent of or in conjunction with cold-exposure [135, 136]. Together, there is no strong evidence to suggest that these bioactives have any impact on enhancing BAT activity which may translate into body weight loss in humans.

### Environmental

#### Cold temperature

##### Evidence in rodents

Cold exposure activates the SNS releasing norepinephrine that binds to β3ADRs, and subsequently activating downstream protein kinase A (PKA) leading to increased Ucp1 activity [3, 137]. HFD feeding impairs this mechanism by altering vagal nerve transmission and decreasing SNS activity [138]. Cold exposure can additionally sensitize transient receptor potential melastatin 8 (TRPM8) channels located on adipocytes to activate PKA signalling [137]. By activating TRPM8 with agonists such as menthol, HFD fed mice are protected against obesity and glucose intolerance [139]. Moreover, deletion of TRPM8 induces obesity and reduces fatty acid oxidation in mice housed in mild cold temperatures [140]. The activation of BAT in response to cold exposure can be attributed to various pathways and present different therapeutic strategy to sustain thermogenic activity.

##### Evidence in humans

**FIGURE 1:**
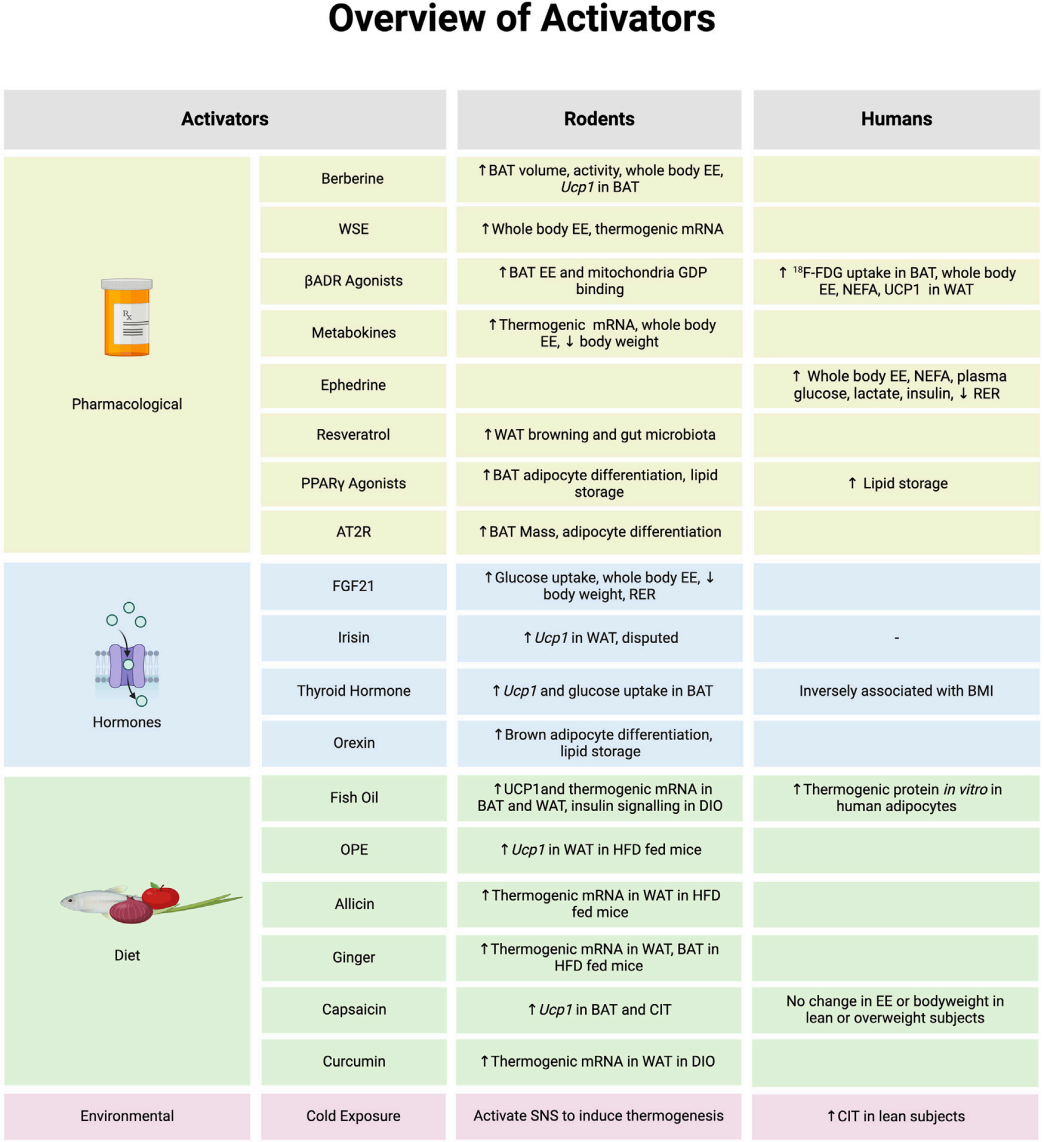
An overview of molecules that activate brown adipose tissue (BAT) or browning of white adipose tissue (WAT) and evidence to support in biological model of rodents or humans. *Note: the evidence summarized in each section represents data provided from both *in vitro* and *in vivo* models in rodents and humans. Abbreviations: Angiotensin type 2 receptor (AT2R), Beta-adrenergic receptor (βADR), Cold-induced thermogenesis (CIT), Diet induced obesity (DIO), Energy expenditure (EE), Fibroblast growth factor 21 (FGF21), Fludeoxyglucose (^18^F-FDG), Guanosine diphosphate (GDP), High fat diet (HFD), Non-esterified fatty acid (NEFA), Onion peel extract (OPE), Peroxisome proliferator-activated receptor γ (PPARγ), Respiratory exchange ratio (RER), Sympathetic nervous system (SNS), *Withania somnifera* extract (WSE), Uncoupling Protein 1 (Ucp1). Image created in Biorender.

In a retroactive prospective study by Becher et al, across 52,000 patients imaged between 2009 and 2018 using ^18^F-FDG PET/CT only 9.7% had detectable BAT [9]. Similarly, across 11,000 patients imaged in another study, only 8% had detectable BAT [141]. Controlling for an effect of cold exposure can only apply to a small cohort of individuals who have BAT, and so far, no RCTs have evaluated any effective changes to BAT activity which could lead to weight loss. Cold induced thermogenesis (CIT) is compromised in morbidly obese and obese subjects compared to lean counterparts, suggesting that BAT activity is highly dependent on body fat ratio and overall weight [142–145]. Moreover, to measure the impact of casual cold-exposure in humans, young healthy males with a history of winter swimming in temperatures between 1°C and 9°C were assessed for CIT responses following intermittent thermal and cooling sessions compared to controls [146]. Winter swimmers have a greater response to CIT and overall lower core body temperature [146]. Control subjects had greater BAT glucose uptake at thermoneutrality suggesting that BAT is active to maintain core body temperature in adults [146]. Acute (1h) cold exposure for 7 days can reduce skeletal muscle shivering response and upregulate non-shivering thermogenesis in healthy adult males (aged 20–29 years old), however, this effect was not due to changes in whole body fuel selection [147]. These data demonstrate that routine cold-exposure influences thermogenesis and may therefore be exploited to rescue BAT activity in obese individuals. We have summarized BAT activators in Figure 1.

## Inactivators of brown adipose tissue

### Hormones

#### Glucocorticoids

##### Evidence in rodents

Glucocorticoids (GCs) are stress induced steroid hormones produced by the adrenal glands that cause rise in blood glucose levels and adipogenesis, ultimately aiming to maintain sufficient glucose supply to the brain [148]. GCs have been described as inhibitors of BAT and browning activity due to their nature to induce lipid accumulation at high concentrations [7]. In male Wistar rats, corticosterone (active GC in rodents) treatment promotes BAT remodeling towards a WAT phenotype (known as “whitening”) and reduces *Ucp1* and *Prdm16* mRNA expression [149]. In HFD fed mice, GCs negatively regulate metabolism by inducing lipolysis and insulin resistance, and lead to greater adiposity by lowering EE and *Ucp1* expression in BAT [150, 150]. Antagonism of GC’s increases PGC-1α content and sustains weight gain in DIO female mice indicating that blocking the effects of GCs may help to prevent unnecessary lipid spillover from the adipose tissues to peripheral tissues such as the liver [151]. GCs also enable whitening by regulating microRNAs (miRNA), which are small non-coding RNA segments that can change gene expression post-transcriptionally. miR-27b is highly expressed in WAT depots of HFD fed mice [152], and is potentiated by GC exposure to induce adipocyte whitening. Inhibiting miR-27b promotes browning by increasing UCP1 and PRDM16 content in WAT [153, 154]. β-adrenergic stimulation of BAT might effectively counteract the obesity-related effects of GCs and maintain thermogenic activity. In mice subjected to 4 weeks of cold exposure (13°C) and treated with corticosterone (50 μg/ml), BAT mass is preserved accompanied with an enhanced UCP1 protein expression compared to mice housed at room temperatures (22°C) [155]. The exact mechanisms behind how GCs induce whitening in BAT are not fully elucidated. However, Luijten et al demonstrated that GC-induced change in BAT lipid composition is not dependent on the Ucp1 signaling pathway [156].

##### Evidence in humans

In humans, excessive GC levels lead to adipose tissue expansion, impaired appetite and increased risk of diabetes [157]. Around 80% of GC users exhibit weight gain [158]. Patients with chronically excessive levels of GC, known as Cushing’s syndrome (CS), are at greater risk for dyslipidemia, diabetes and obesity due to the impact of GC on lipid and glucose metabolism. Overweight subjects with CS have a negative correlation between cortisol and UCP1 protein compared to controls suggesting that excess GC reduces BAT function [159]. GCs in BAT of obese populations is less explored, however studies have demonstrated differences in acute and chronic GC treatment effects [160–162]. In healthy males, acute GC increases glucose uptake and EE of BAT under cold exposure. However, upon retrospective assessment of chronic GC treatment lasting 2 weeks, a reduction in BAT mass is observed, which correlates with decreased BAT activity [160]. Dosage of GC may also influence the effect on BAT as evident from reduced CIT following mild cold exposure (19°C) in healthy adults treated with a low dose of prednisone (15 mg/d) for 1 week. However, no such changes are observed with high dose prednisone (40 mg/d) for 1-week after cold-exposure (10°C) despite having greater EE [161, 163]. Increase in EE in high-dose prednisone treated males may be attributed to skeletal muscle calcium cycling as prednisone can stimulate calcium cycling genes [161]. While evidence regarding the influence of acute GC treatment in BAT function is diverse in humans, prolonged exposure to GC reduces thermogenic capacity and impairs BAT function. This may be due to differences in dosing and unstandardized treatment regimens.

#### Aldosterone

##### Evidence in rodents

Aldosterone is a mineralocorticoid hormone produced by the adrenal glands. Concerning BAT activity, aldosterone (100 nM) stimulation of the mineralocorticoid receptor (MR) downregulates *Ucp1* expression in brown adipocytes. This effect occurs through the inhibition of retinoid X receptor (RXR) and retinoic acid receptor (RAR) transcription factors located along the *Ucp1* gene [164]. Administration of mineralocorticoid receptor antagonists (MRA) including finerenone, have been successful in preserving thermogenic markers by increasing UCP1, AMPK, and ATGL content in brown pre-adipocytes, indicating a greater fat mobilization and promotion of thermogenic pathways [165]. Similarly, in healthy and HFD fed mice, MRAs increase both iBAT density and thermogenic browning gene expression of *Ucp1*, *Prdm16*, *Pgc1-*α and *Cidea* [165–167]. In contrast, aldosterone deficiency in HFD fed mice neither prevent obesity nor alter insulin efficiency. However, it moderately alleviates white adipocyte dysfunction, as indicated by increased plasma adiponectin levels and reduced macrophage infiltration [168]. Collectively, these findings indicate that blocking MR activity may improve BAT thermogenic capacity, but no studies measure the direct impact of MRA treatment on the ability to withstand colder environments.

##### Evidence in humans

According to a study by Rossi et al, comprising of male (n = 56) and female (n = 44) obese/overweight patients with hypertension, there is a positive association between BMI and plasma aldosterone concentrations [169]. MR expression in sWAT and vWAT was greater in obese populations suggesting an increased risk for adipocyte inflammation [170], however this study only provided analysis in a sample size of 7, without direct assessment of adipocyte dysfunction or inflammation. In lean populations, MRA increased thermogenic activity indicated by ^18^F-FDG/PET-CT imaging and BAT mass in supraclavicular regions compared to placebo control [171]. However, MRA treatment was only for 2 weeks and disproportionate sampling from males and females along with small sample size warrants further investigations [171].

#### Androgens

##### Evidence in rodents

Androgens, such as testosterone and dihydrotestosterone, are steroid hormones that play a role in the development of male reproductive system and characteristics. Several studies demonstrate an association between androgen activity and reduced thermogenic capacity. In healthy castrated male mice, WAT thermogenic capacity is high and sensitive to cold-induced browning [172], however, increasing concentrations of testosterone (10^-9^–10^-7^ M) reduced *Ucp1* expression in differentiated adipocytes obtained from cervical, interscapular and auxiliary BAT in mice [173]. Androgen receptor knockout male mice fed either regular chow or HFD have reduced *Ucp1* gene expression and greater weight gain with vWAT accumulation, suggesting that androgen receptor signalling contributes to fatty acid uptake in adipose tissues [174, 175]. These studies provide conflicting literature on androgens and their receptor signaling, and therefore, more studies are needed to determine if androgen receptor agonism or antagonism at the level of the adipose tissue is needed to improve BAT function.

##### Evidence in humans

**FIGURE 2:**
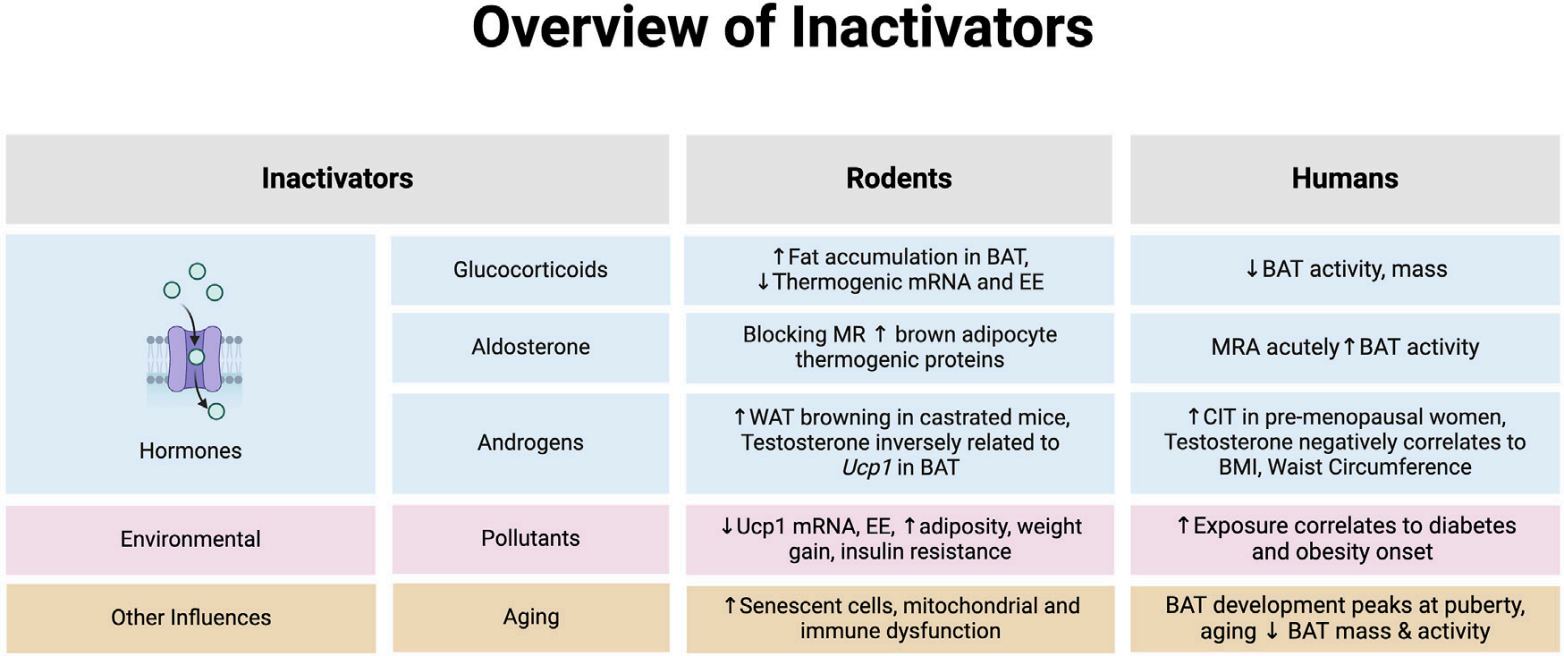
An overview of hormones that inhibit brown adipose tissue (BAT) or browning of white adipose tissue (WAT) in rodents and humans. *Note: the evidence summarized in each section represents data provided from both *in vitro* and *in vivo* models in rodents and humans. Abbreviations: Body mass index (BMI), Cold-induced thermogenesis (CIT), Energy expenditure (EE), Mineralocorticoid receptor (MR), Mineralocorticoid receptor antagonist (MRA). Image created in Biorender.

A longitudinal analysis by Gapstur et al showed reductions in testosterone levels with age and higher greater BMI and waist circumferences [176]. Studies in women with polycystic ovarian syndrome have reported conflicting results on a correlation between obesity and testosterone. Additionally, no association between testosterone and supraclavicular skin temperatures were noted [177–179]. Recent studies have found that CIT is greater in premenopausal compared to post-menopausal women, which could imply that estrogen is linked to improved thermogenic activity [180, 181]. Stronger evidence is required to determine the independent role of androgen signaling in human populations as results are inconclusive so far. BAT inactivators in rodents and humans are summarized in Figure 2.

## Environmental inhibitors

### Environmental pollutants

#### Evidence in rodents

In recent years, environmental pollutants such as pesticides, and air pollution have become associated with negative impacts on human health and have contributed to the development of obesity [182]. Pesticides such as deltamethrin pose harmful effects on brown adipocytes by reducing expression of *Ucp1* and cAMP activity. Interestingly, however, in male mice, deltamethrin had no impact on body composition and, at a low dose of 0.01 mg/kg/day, actually improved insulin sensitivity and energy expenditure [183]. Recently, 34 chemicals were identified due to their abundance in food product and packaging concluded that chlorpyrifos (CPF), a organophosphate pesticide commonly found in fruits, vegetables, grains, and meats was linked to reductions in *Ucp1* mRNA expression and mitochondrial respiration in immortalized brown adipocytes [184]. Concentrations of CPF as small as 1 pM reduced mitochondrial membrane potential, activity of complex IV in the electron transport chain, and RNA transcripts of genes involved in fatty acid oxidation [184]. In HFD fed mice, CPF increased weight gain and adiposity of WAT, reduced energy expenditure and inhibited diet-induced thermogenesis [184]. CPF also reduced cAMP activity in brown adipocytes, suggesting that CPF may also impair sympathetic activation of BAT thermogenesis [184]. Pesticides such as dichlorodiphenyltrichoroethane (DDT) have been previously associated with metabolic disruption and when exposed perinatally to female mice, lead to reduced energy expenditure, glucose intolerance and increased adiposity [185]. Additionally, when placed on a HFD, offspring develop insulin resistance and reduced *Ppargc1a* expression in BAT thus impairing thermogenesis [185]. Various other pesticides including permethrin and bifenthrin promote weight gain, insulin resistance, inflammation and lipid accumulation in adipose tissue, however direct effects on BAT thermogenesis have yet to be determined [186]. Air pollutants also induce changes to BAT in male mice by increasing reactive oxygen species, reducing expression of UCP1 protein expression, and BAT mitochondrial area and count [187].

#### Evidence in humans

To date, studies have not investigated the impact of pesticides in human BAT; however, many studies have documented the association between pesticides and obesity. Research on farmers has confirmed greater incidences of diabetes and insulin resistance linked to routine exposure to organophosphate pesticides, including CPF [188–190]. Similarly, the use of pesticides has also been correlated with a greater risk of obesity among farmers in Thailand [191]. However, causality of these links has yet to be established.

## Other influencers of BAT

### Aging

Effects of aging on BAT is largely attributed to changes in immune and senescent cell activity as well as mitochondrial dysfunction. With aging, the immune system becomes more unregulated, and there is an increase in pro-inflammatory M1 macrophage activation within adipose tissue [192]. Additionally, there is an increased presence of senescent immune cells in BAT which damage adipose tissue by secreting inflammatory cytokines. Senescent immune cells reduce expression of thermogenic markers such as *Ucp1* and *Ppargc1a*, and also downregulate gene expression of adipose RNA binding motif 3 (RBM3) which prevents sympathetic innervation of the BAT [193]. Senescent T cells also promote BAT whitening in aging mice by secreting IFN- γ, which inhibits brown adipocyte differentiation [194]. Mitochondrial dysfunction increases with age and leads to a greater oxidative stress. Cui et al found that 20-month-old mice have less expression of BAT thermogenic genes including *Ucp1* and *Ppargc1a* in contrast to 6-week-old mice [195]. These changes in BAT were accompanied by a decrease in antioxidants, indicating higher age-related oxidative stress. Inducing oxidative stress using hydrogen peroxide in brown adipocytes also reduced the quantity and morphology of mitochondria; however, these effects were rescued by antioxidant treatments [195]. In humans, similar declines in BAT mass can be observed due to age, with some studies observing that the decline is more prevalent in males than females [9, 196]. Intriguingly, a puberty-related rise in BAT mass has been described in children over the age of 10, suggesting that BAT development peaks with sexual maturity and musculoskeletal development, coinciding with an increase in growth and sex-related hormones [197–199]. However, despite this rise, a decline in BAT activity persists into adulthood, likely attributed to greater body fat accumulation. The mechanisms underlying the regulation at the level of brown adipocytes remain unclear [200].

### Biological sex and sex hormones

Sex based differences in BAT morphology and activity deserve attention. Although the majority of studies have included males, there are notable differences in BAT function between males and females [201]. Female rats have been shown to have greater BAT mass, mitochondrial content, and *Ucp1* expression compared to male rats [202, 203]. PET/CT scanning has shown that females may have lower BAT volume but similar activity compared to male [204]. Conversely, other studies have shown that females have greater detectable BAT than males [205, 206]. Sex based differences in BAT presence are age-related, with higher BAT activity in females at younger ages. However, this difference becomes obsolete in post-menopausal women, indicating that changes in sex hormone levels may contribute to BAT function [201]. Recently, Blondin et al, [181] has demonstrated that BAT oxidative metabolism and glucose uptake is greater in premenopausal women in comparison to postmenopausal women but the change in BAT tissue radiodensity, which is an indirect lipid content in BAT was not altered between groups of women. Transcription of the batokine BMP8B, which aids in modulating BAT for thermogenesis, is promoted in female mice with estradiol 2 (E2, the main circulating form of estrogen) treatment, whereas ovariectomy drastically decreases BMP8B expression in female mice [207], and decreases thermogenic activity and *Ucp1* expression [208]. The mechanisms of estrogen induced increases in thermogenic activity may be through the activation of the estrogen receptor α leading to subsequent norepinephrine stimulated lipolysis. In contrast, testosterone levels lowers the rate of lipolysis [209]. Estrogen can also influence BAT activity via sympathetic nervous system stimulation to increase UCP1 protein expression and thermogenic activity [210]. The *in vivo* effects of androgens on BAT activity are less clear. Removal of testes, the primary location of testosterone production, increases UCP1 protein expression in BAT implying a blunting effect of testosterone on BAT thermogenic ability [211]. However, it is difficult to determine if changes in BAT function are directly mediated by testosterone signalling or by the conversion of testosterone to E2 in the tissue that may be modulating BAT activity [201]. It is important to consider the complexities and importance of sex-based genetic and hormonal influences on BAT function when investigating potential BAT activators to combat obesity, to meet the needs of all individuals who could benefit from these therapies. A comprehensive discussion of the effects of biological sex on BAT function, morphology, and growth has been discussed elsewhere [201, 212, 213].

### Exercise

Evidence that exercise influences BAT activity is more prevalent in rodents than in humans. In obese male mice, 4 weeks of daily aerobic exercise increased BAT mass while also upregulating genes involved in glucose and lipid metabolism, however only serum glucose levels were reduced following training, whereas serum lipid and cholesterol were unchanged [214].

Exercise can also stimulate browning of WAT. Male Swiss rats that underwent 8 weeks of aerobic or resistance training [215], had significantly higher Ucp1 protein*, Ppargc1a,* and *Cidea* gene expression in both inguinal and retroperitoneal WAT [216]. Moreover, swim training in Sprague-Dawley rats fed a HFD demonstrate reductions in weight but does not alter the thermogenic profile in BAT. Instead, it promotes myogenic protein markers, suggesting that BAT may adopt a muscle-like oxidative function during exercise rather than a thermogenic function [216]. Conflicting evidence on the influence of exercise on BAT has emerged, as studies in humans fail to replicate findings seen in rodents. Recent research in sedentary humans demonstrates that exercise has no impact on BAT activation [217]. In the ACTIBATE trial, no changes in BAT volume or F-FDG uptake were identified across control, moderate-exercise, and vigorous-exercise groups following 24 weeks of combined endurance and resistance exercises [217]. Moreover, various studies in both male and female endurance athletes report lower BAT activity and volume compared to non-athletes counterparts, challenging the notion that BAT and exercise are complementary [218, 219].

Exercise also stimulates the release of hormones or activating factors, referred to as exerkines, that can influence BAT activity. Of note is AMPK, a key regulator of skeletal muscle metabolism. In relation to BAT, AMPK activation increases *Ucp1* expression in differentiated brown adipocytes derived from mice undergoing 4 weeks of voluntary aerobic training [220]. Exercise also improves efficiency of exerkines such as FGF-21 in obese mice by increasing the expression of KLB receptors in BAT [221]. However, not all exerkines affect BAT as seen with irisin. After acute swimming interventions, male mice show increased FDNC5 but no changes in *Ppargc1a*, *Ucp1* gene expression, or irisin protein levels in BAT [222]. In humans, the effects of exerkines are more varied. A recent study assessing 16 exerkines, including FGF-21, lactate, and irisin found that endurance activities increased FGF-21, reduced lactate, but failed to detect irisin or measure any changes [223]. This study, which had limitations due to its small sample size and inclusion of only female participants, concluded that exercise altered the circulation of exerkines other than irisin [223]. However, only changes in lactate levels were associated with changes in BAT volume [223]. Overall, exercise and BAT activity shows that this relationship is less reproducible in humans than in rodents. Although exercise and BAT thermogenesis have positive effects on energy expenditure and metabolism that result in weight loss, they work independently rather than in tandem. The impact of exercise likely outweighs any influence of BAT thermogenesis, as exercise demands a greater utilization of energy substrates and restricts blood flow to adipose tissue overall, whereas it stimulates or maintains muscle mass that makes up the majority of body weight as least in healthy humans.

## Discussion

### Is UCP1 indispensable in regulating BAT activity?

Ucp1 is a key protein involved in facilitating thermogenesis in BAT. However, evidence suggests that Ucp1 is only functional when it is activated. This is evident when mice with 50 times higher amount of Ucp1 are placed on a high fat/sucrose diet. Despite higher UCP1, animals experience weight gain at a similar rate to controls [224]. Only upon injection of β3ADR, mice with higher UCP1 display greater EE, indicating how activation of Ucp1 is key to producing any thermogenic effects. Similarly, *Ucp1* ^−/−^ and WT mice exhibit similar levels of EE and susceptibility to DIO at thermoneutrality. However, with noradrenaline treatment Ucp1 was activated in WT mice leading to greater EE [225]. This highlights that thermogenic activity in BAT does not occur ubiquitously and requires on-going activation of Ucp1. Therapeutic interventions must achieve sustained activation of Ucp1 for pharmacological efficacy. Current options capable of Ucp1 activation, such as βADR agonists, have inherent limitations as they increase heart rate and blood pressure [20].

On the other hand, Ucp1 is dispensable for thermogenesis evident from recent, studies that have identified non-shivering thermogenic processes that act independently of Ucp1 [10, 226], one of these being calcium cycling present in beige adipose tissue. Calcium (Ca^2+^) is released and sequestrated by the sarco-endoplasmic reticulum (SERCA) through SERCA2b in beige adipocytes [10, 227]. This cycling process releases heat as a by-product and utilizes ATP to fuel SERCA2b uptake of Ca^2+^ into SERCA [10]. Ca^2+^ cycling is activated either by β-ADR stimulation [10] or cold-exposure [10, 228], and this process seems to be protective against DIO at thermoneutrality in both WT and *Ucp1* ^−/−^ mice [229].

Another UCP1-independent ATP-dependent mechanism of thermogenesis is creatine substrate futile cycling where phosphocreatine (PCr) is shuttled from the mitochondria towards the endoplasmic reticulum (ER) to be converted to ATP and creatine (Cr) by creatine kinase (CK) [11]. This cycle of ATP production through the PCr/CK cycle, which primarily takes place in beige adipocytes, provides fuel for calcium cycling, as SERCA2b present in the endoplasmic reticulum of cells can utilize ATP to take up Ca^2+^ by the support of cytosolic CK, leading to the dissipation of heat [11]. Kazek et al demonstrated that reductions in creatine induced by β-guanidinopropionic acid were linked to reductions in oxidative metabolism in both iWAT and BAT following β-3ADR agonist treatments [15]. Additionally, they found that among *Ucp1* ^−/−^ mice, those treated with β-GPA had reduced ability to maintain body temperature after cold exposure (4°C) compared to vehicle controls. The absence of creatine kinase B (Ckb), a key regulator of creatine cycling, in mice leads to decreases EE and increased risk of obesity [230]. This shows that in the absence of Ucp1, Cr cycling serves as a compensatory measure to maintain thermogenesis. This is corroborated by adipocyte-selective inactivation of Ckb that diminishes thermogenesis and predisposes to obesity [15]. Recently, demonstrated in mice with adipocyte-selective deletion of either Ucp1 or Ckb are euthermic which worsens cold intolerance making mice hypothermic. This suggests that thermogenic adipocytes use redundant, non-paralogous proteins to maintain body temperature [231]. Although further studies are required for comprehensive understanding, this suggests that while Ucp1 plays a significant role in thermogenesis, it is not indispensable.

### The potential for BAT to mitigate obesity

It is important to consider the availability or “recruitability” of BAT and the amount it contributes to whole body EE when assessing its potential as a therapeutic strategy to manage obesity. We know that BAT is relatively less abundant in human adults, in both lean and obese populations compared to rodents [9]. Early studies in human BAT indicated that CIT (16°C) was significantly reduced in individuals that were obese compared to lean individuals [232]. More recently, when quantifying changes following CIT (16°C), lean males still seem to achieve greater BAT activity with a 17% increase in basal metabolic rate, whereas obese males only experienced an increase by 6% [144]. Among healthy males and females, following mild CIT (15.5°C), BAT only attributed to a modest 15–25 kcal/day increase in EE [233] and another study assessing EE following cold stimulus at 6°C found that BAT only contributed to a 10 ± 5 kcal/day increase in EE [234]. Additionally, the exact contribution of BAT activity to EE is questionable where cervio-thoracic muscles, as observed in PET-CT scans, have indicated greater impacts to cold-induced EE than BAT [234]. Interestingly, no linear relationship between BAT and whole-body EE has been indicated, and BAT contributions only represented 1% of the total whole-body changes in EE after CIT [234]. Although BAT can contribute towards whole body EE, which is especially apparent in rodents, the evidence thus far is insufficient to contribute weight reduction in obese human populations. It is also important to explore the benefits of BAT activation beyond solely increasing EE to aid in weight loss. BAT activation may provide protection against metabolic dysregulation such as hyperglycemia and hyperlipidemia associated with obesity. BAT activation using CL, 316243 (β3ADR agonist) over 10 weeks in *E3L and CETP* mice that closely resemble human lipoprotein metabolism robustly lowered plasma TG and total cholesterol levels while maintaining identical food intake [235]. These mice also showed significant reduction in atherosclerotic lesion size after 10 weeks of treatment further demonstrating the protective effects of highly functional BAT. Cold exposure increases glucose uptake in BAT [236, 237] even in fasted rats with low insulin levels [238] indicating sympathetic induced glucose uptake. While glucose is not thought to constitute much of the thermogenic substrate, it may be used in the intracellular synthesis of triglycerides for thermogenesis [3]. In metabolically healthy men with functional BAT, cold exposure significantly increased glucose uptake into BAT, resting energy expenditure, and glucose and lipid oxidation [239]. However cold exposure has little effect in healthy individuals without detectable BAT even during cold exposure. Given that the prevalence of BAT is low, maximizing tissue activity is challenging and may not have an overwhelming effect on energy expenditure to offset obesity, and might not be responsive across all individuals and patient populations. Thus, we conclude that it is not yet adequately reported in humans that BAT is an effective therapeutic target to induce enough weight loss to protect against the adverse effects of obesity but may be a tissue that can detect and influence energy metabolism.
